# Recent advances in management of phaeochromocytoma

**DOI:** 10.1186/1687-9856-2015-S1-O7

**Published:** 2015-04-28

**Authors:** Nalini S Shah

**Affiliations:** 1 Dept of Endocrinology, K E M Hospital, Mumbai, India

## 

Pheochromocytoma (PCC) and paraganglioma (PGL) are the neuroendocrine tumours that arise in adrenal medulla or extra adrenal sympathetic and parasympathetic paraganglia. Paraganglia are small organs that mainly consist of neuroendocrine cells derived from the embryonic neural crest that have the ability to synthesize and secrete catecholamines. Sympathetic PGLs are usually located in the chest, abdomen, or pelvis while parasympathetic PGLs are distributed along parasympathetic nerves in the head, neck, and upper mediastinum, also referred to as head and neck PGL (HNP). PCC/PGLs can be either functional (sympathetic) or non-functional (parasympathetic) based on catecholamine synthesis and secretion.

In children diagnosed with hypertension, up to 1.7% have a catecholamine-secreting neoplasm. The majority of these tumours in childhood are PCC/PGLs. Incidence rates of PCC/PGLs are estimated at 0.3 cases per million per year or less. Approximately 10 –20% of these cases are diagnosed during childhood. Approximately half of the apparently sporadic PCC/PGLs that present in patients younger than 18 years are due to an identifiable germline mutation and this increases up to 70% in children less than 10 years of age. Hereditary tumor syndromes associated with PCC include Von Hippel-Lindau (VHL) disease, multiple endocrine neoplasia 2 (MEN-2), the familial PGL syndromes and rarely, neurofibromatosis (NF) type 1. Recently, many other genes are found to be involved in pathogenesis of PCC/PGLs. Hereditary PCC/PGLs are often multifocal and, in the case of PCC, frequently bilateral.

Children usually present becauseof symptomatic catecholamine hypersecretion or, less often, due to tumor mass effects (e.g. pain), or as an incidental radiographic finding, or because of family screening. At present, the diagnostic test of choice is the measurement of fractionated plasma and/or urine metanephrines (metanephrines and normetanephrines). A four-fold increase above the reference range is associated with an almost 100% probability of the presence of a catecholamine-secreting tumor.

^123^I-metaiodobenzylguanidine(MIBG) is a highly specific test that can confirm the catecholamine-secreting nature of a tumor, localize tumors not seen with cross-sectional imaging, and identify other sites of disease as well as metastasis. Because MIBG testingis not 100% sensitive, particularly in some genetic mutations, recently other functional scans have been studied in the evaluation of PCC/PGL: ^18^F fluorodihydroxyphenylalanine (DOPA) positron emission tomography (PET), ^18^F fluorodopamine (FDA) PET,^18^F fluorodeoxyglucose (FDG) PET.

Surgical resection is the mainstay in the treatment of PCC/PGLs. It should be done after adequate alfa and beta adrenergic blockade to minimize the hemodynamic fluctuation during surgery. Laparoscopic adrenalectomy is the preferred procedure for most PCC. Laparotomy should be considered in patients with large PCC and/or a concern for underlying malignancy based upon the clinical presentation or radiographic appearance of the tumor. In the setting of bilateral PCC, cortical-sparing procedures should be considered for the adrenal with the least tumor bulk. The cortical-sparing approach is particularly attractive in young children and children at risk for noncompliance with lifelong glucocorticoid and mineralocorticoid replacement. The surgical approach for removal of a PGL depends upon the location of the tumor but in selected cases can also be performed laparoscopically.

Patients with unresectable malignant tumors or distant metastatic disease can usually be treated symptomatically with adrenergic blockade. Radiation therapy or radiofrequency ablation can help with symptomatic metastatic disease, and IV bisphosphonates can be considered bony metastases, particularly in patients with bone pain or lesions that increase the risk of pathological fracture. Systemic treatment modalities are only palliative in nature and include ^123^I-MIBG therapy, peptide receptor radiotherapy (PRRT) and chemotherapy. PCC/PGLs can have unpredictable behavior and metastasize late in the clinical course. In addition, children in particular are at risk for the development of metachronous tumors. This makes long-term follow-up mandatory in children with PCC/PGL.

